# Low Carbohydrate, High Fat Diet Alters the Oral Microbiome without Negating the Nitrite Response to Beetroot Juice Supplementation

**DOI:** 10.3390/nu15245123

**Published:** 2023-12-16

**Authors:** Louise E. Cato, Alannah K. A. McKay, Joanna E. L’Heureux, Anni Vanhatalo, Andrew M. Jones, Christopher D. Askew, Gary J. Slater, Louise M. Burke

**Affiliations:** 1 School of Health, University of the Sunshine Coast, Maroochydore, QLD 4558, Australia; caskew@usc.edu.au (C.D.A.); gslater@usc.edu.au (G.J.S.); 2 Mary MacKillop Institute of Health Research, Australian Catholic University, Melbourne, VIC 3000, Australia; alannah.mckay@acu.edu.au (A.K.A.M.); louise.burke@acu.edu.au (L.M.B.); 3 University of Exeter Medical School, University of Exeter, Exeter EX1 2LU, UK; j.lheureux3@exeter.ac.uk (J.E.L.); a.vanhatalo@exeter.ac.uk (A.V.); a.m.jones@exeter.ac.uk (A.M.J.)

**Keywords:** nitric oxide, low carbohydrate high fat, oral microbiome, nitrate, ketogenic

## Abstract

A low carbohydrate, high fat (LCHF) diet in athletes increases fat oxidation but impairs sports performance, potentially due to impaired exercise economy. Dietary nitrate supplementation can improve exercise economy via an increase in nitric oxide production, which is initiated by the reduction of nitrate to nitrite within the oral cavity. This reaction is dependent on the presence of nitrate-reducing oral bacteria, which can potentially be altered by dietary changes, including a LCHF diet. This study explored the effect of a LCHF diet on the oral microbiome and subsequent changes to plasma nitrite concentration following nitrate supplementation. Following five days of LCHF or high carbohydrate (HCHO) control dietary intervention, highly trained male race walkers consumed 140 mL beetroot juice containing 8.4 mmol nitrate; they then provided (a) blood samples for plasma nitrate and nitrite analysis and (b) saliva samples for 16S rRNA sequencing of the oral microbiome. The LCHF diet (n = 13) reduced oral bacterial diversity and changed the relative abundance of the genera *Neisseria* (+10%), *Fusobacteria* (+3%), *Prevotella* (−9%), and *Veillonella* (−4%), with no significant changes observed following the HCHO diet (n = 11). Following beetroot juice ingestion, plasma nitrite concentrations were higher for the LCHF diet compared to the HCHO diet (*p* = 0.04). However, the absence of an interaction with the trial (pre–post) (*p* = 0.71) suggests that this difference was not due to the dietary intervention. In summary, we found an increase in plasma nitrate and nitrite concentrations in response to nitrate supplementation independent of diet. This suggests the oral microbiome is adaptive to dietary changes and can maintain a nitrate reduction capacity despite a decrease in bacterial diversity following the LCHF diet.

## 1. Introduction

Interest in the ketogenic low carbohydrate, high fat (LCHF) diet has re-emerged for endurance athletes in recent years, principally due to its capacity to substantially increase the rate of fat oxidation during exercise [1]. Despite a doubling of the rate of fat oxidation during exercise, and even in the case of training-induced adaptations such as an increase in VO2max, the LCHF diet has been consistently associated with an impairment, rather than an improvement, of performance in high-intensity endurance events [2,3,4,5]. This finding has been attributed to a reduction in exercise economy (i.e., increased oxygen cost of exercise) associated with the stoichiometry of fat oxidation [6,7]. Given that exercise economy is a key determinant of endurance exercise performance [8], strategies that may counteract this deleterious effect of a LCHF diet are of interest. Dietary nitrate supplementation is one strategy known to improve exercise economy [9] and has been proposed as a possible method to ‘rescue’ the reduction in exercise economy observed in response to a LCHF diet [10]. However, to date, this has not been verified.

Nitric oxide (NO) can be produced via two different pathways: the well-recognised oxidation of L-arginine or the reduction of endogenous nitrate [11], e.g., through dietary intake or supplementation. This latter pathway becomes increasingly important under scenarios in which the traditional NO pathway is impaired, such as in the presence of the local acidity and hypoxia associated with sustained high-intensity endurance exercise. The nitrate-NO reduction pathway is dependent on the conversion of nitrate to nitrite by anaerobic bacteria in the oral cavity [12]. The disturbance of oral nitrate-reducing bacterial activity, such as following the use of anti-bacterial mouthwash, is associated with a blunted increase in plasma nitrite concentration [13], and differences in the oral microbiome may provide one explanation for the inter-individual variation in the physiological and performance responses to nitrate supplementation [12].

The oral microbiota is a complex ecosystem that has large variability in the diversity and abundance of bacterial species [12,14,15,16], as well as differences in nitrate reduction activity. Numerous factors are known to influence this ecosystem, including bacterial genetics, environmental factors, and host factors such as gender [17], age, diet, and ethnicity [18,19]. There is preliminary evidence that exposure to a LCHF diet is associated with changes to the oral microbiome [20], and these changes may have implications for net oral nitrite production following bolus nitrate ingestion. A decrease in background concentrations of plasma nitrite from dietary sources may contribute to the reduction in exercise economy seen with the LCHF diet [2,5]. It may also explain previous observations that a high fat diet was associated with a reduced conversion of plasma nitrate to nitrite following potassium nitrate supplementation [10]. We hypothesised that the adaptation to a LCHF diet would alter the oral microbiome, impairing its nitrate-reducing capacity both in a control situation and in response to acute nitrate supplementation.

## 2. Materials and Methods

This study formed part of a larger investigation exploring the effect of short-term exposure to the LCHF diet on metabolism and performance in elite athletes, with data collected over two separate training camps. The recruitment of highly-trained Tier 3 (National level) to Tier 5 (World Class) race walkers [21] and strict control of the dietary interventions provided an ideal opportunity to explore changes to the oral microbiome and nitrate-reducing capacity in response to dietary intervention. Ethics approval for this study was obtained through the Human Ethics Committee of the Australian Institute of Sport (AIS) with ethics approval numbers 20181203 (study 1) and 20191102 (study 2). All participants provided written informed consent after receiving comprehensive oral and written details of the protocol.

### 2.1. Overview of Study Design

**Figure 1:**
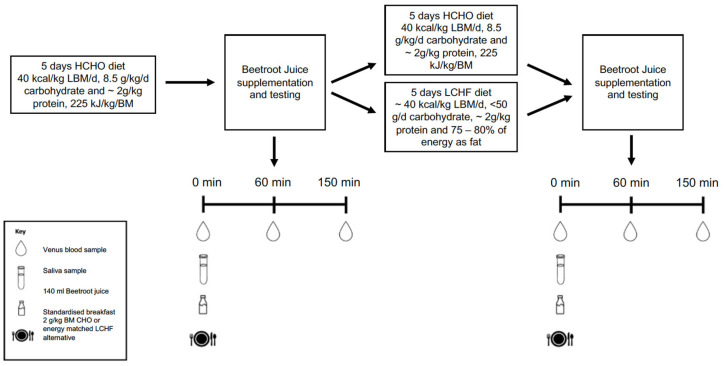
Overview of laboratory studies undertaken before and after 5 days of the high carbohydrate (HCHO) or low carbohydrate, high fat diet (LCHF).

A non-randomised, parallel group study design was used across the two separate training camps to collect 24 data sets from highly-trained race walkers. Due to difficulties in blinding athletes to dietary interventions, diets were assigned according to athlete beliefs on the potential performance implications, with justification for this methodology provided elsewhere [2]. Dietary standardisation consisted of five days of either a high carbohydrate (HCHO) or LCHF diet, with the HCHO diet acting as a control. All athletes initially undertook the HCHO diet for five days to establish baseline values, after which plasma and saliva samples were collected to determine nitrate response and oral microbiome composition. Another five days of dietary standardisation followed, with athletes allocated either a LCHF or HCHO diet, with plasma and saliva samples again being collected at the end of the dietary intervention (Figure 1).

### 2.2. Participants

Highly competitive (Highly Trained/National level, Elite/International level, and World Class [21]) male race walkers were recruited for this study, which was run over two separate three-week training camps at the Australian Institute of Sport (AIS) in Canberra in 2019 and the Australian Catholic University (ACU) Melbourne campus in 2020. These training camps represented baseline preparation for the 2019 and 2020 International Association of Athletics Federation (IAAF) race walking seasons. Seventeen individual athletes contributed to the study with seven athletes participating in both training camps (six crossing over dietary conditions), giving a total of 24 complete data sets.

### 2.3. Dietary and Training Intervention

Each camp consisted of a three-week continuous training block, during which a high carbohydrate (HCHO) diet (~40 kcal/kg LBM/d, 8.5 g/kg/d carbohydrate, ~2 g/kg protein, and 20% of energy as fat) was implemented for five days to establish a baseline, followed by five days of either an isoenergetic low carbohydrate, high fat (LCHF) diet (~40 kcal/kg LBM/d energy, <50 g/d carbohydrate, ~2 g/kg protein, and 80% of energy as fat) or a repeat of the HCHO diet to act as a control. During this period, athletes undertook supervised training sessions incorporating race walking, resistance training, and cross training, which were described via daily training and wellness logs.

All food and fluids were provided to athletes for the duration of the two training camps and consumed under the supervision of accredited sports dietitians. Menu construction and preparation of meals and snacks were undertaken by a team of trained chefs, food service dietitians, and sports dietitians. Each meal plan was adjusted for individual food preferences and requirements to ensure compliance while adhering to the energy and macronutrient targets. Nutrition support for training sessions was included within the daily nutrition targets for each dietary intervention. Athletes were asked to maintain food diaries and record any deviations to the prescribed diet, which were checked nightly by trained sports dietitians. Further details on the dietary interventions and methodology employed to ensure strict dietary control are available elsewhere [22].

### 2.4. Dietary Nitrate

An estimate of daily nitrate intake (mg/day) was obtained using previously established methodology [23] and with the use of a recently developed, comprehensive database of the nitrate content of major food sources [24]. The database, compiled using a systematic approach, contains 4254 records sourced from 256 references to provide data on 178 vegetables as well as 22 herbs and spices from 56 countries. The median nitrate value (mg/kg) for each vegetable was obtained from the database and multiplied by g/day (from a weighted food diary) of each food included in each individualised diet to obtain total daily nitrate consumption.

For non-vegetable items, an estimate of nitrate concentration (mg/kg) was derived using data from 3 published sources [24,25,26]. Where food items appeared in multiple databases, the FSANZ value (lower bound mean) was used. If a nitrate value could not be found, or a suitable substitute was not available, the food was assigned a value of 0.

For recipes used in the study diets, each ingredient was assigned a nitrate value based on either weight or % total contribution to the food (e.g., pesto sauce).

### 2.5. Test Procedures

Athletes presented to the laboratory in an overnight-fasted, rested state following five days of dietary standardisation (HCHO or LCHF diet) and were instructed not to use mouthwash for the duration of the study or to brush their teeth on the study morning. On arrival, baseline blood and saliva samples were collected in the fasted state, prior to consumption of a standardised breakfast for the HCHO diet (HCHO 39 kJ/kg, 0.2 g/kg protein, 2.1 g/kg carbohydrate, and 0.1 g/kg fat), and energy matched for the LCHF diet (40 kJ/kg, 0.6 g/kg protein, 0.6 g/kg carbohydrate, and 0.6 g/kg fat). Both baseline and intervention breakfasts included 2 × 70 mL commercially available nitrate-rich beetroot juice shots (Oz Beet-it, James White Drinks, Ipswich, UK), containing 4.2 mmol nitrate per bottle. For the duration of the data collection, athletes rested quietly with no further food or fluid consumed. 

Saliva samples were obtained at baseline via passive saliva collection according to manufacturer instructions using the OMNIgene saliva collection kit (Omnigene-Oral OM 501, DNA Genotek Inc, Ottowa, ON, Canada). Venous blood samples were collected into 3 mL lithium heparin-coated tubes at baseline (T0) and 60 (T60) and 150 (T150) minutes post beetroot juice ingestion. Samples were subsequently centrifuged at 2200 G, 4 °C for 10 min, aliquoted, and subsequently frozen at −80 °C until batch analysis for nitrate and nitrite was conducted at a later date. Plasma nitrate was analysed spectrophotometrically after deproteinisation by centrifugation through a 30 kDa molecular weight filter (Pall Nanosep, Pall Corporation, Show Low, AZ, USA), and plasma nitrite was quantified by gas phase chemiluminescence (Sievers nitric oxide analyser 280i, Analytix Ltd., Durham, UK).

Saliva samples were placed in a 1.5 mL microcentrifuge tube containing 600 µL of cell lysis solution (QIAGEN). Samples were stored at −80 °C until extraction of oral bacteria DNA using a QIAGEN Gentra Puregene Buccal Cell kit in accordance with the manufacturer’s instructions (Qiagen, Germantown, MD, USA). Library preparation and PCR amplification of the 16S region were completed using NEXTflex 16 S V1-V3 Amplicon-Seq Kit and Barcode primers 1–80 (Bioo Scientific, Austin, TX, USA). The oral bacteria were sequenced using v3 MiSeq reagents and the paired-end 300 base pair MiSeq Illumina platform (Illumina, San Diego, CA, USA). FASTQ nucleotide sequences were trimmed using Trim-Galore! wrapper script (Kruger F. Trim-Galore!, Version 0.6.5, accessible at http://www.bioinformatics.babraham.ac.uk/projects/trim_galore/ (accessed on 7 May 2023)).

### 2.6. Statistical Analysis

Plasma nitrite and nitrate data were analysed using a General Linear Mixed Model in R Studio (v3.2). Fixed effects for diet, pre–post intervention trial, and timepoint (T0, T60, T150) were used, with random intercepts for camp and subject identification included. Visual inspection of residual plots and QQ plots clearly showed data were not normally distributed; therefore, data were log-transformed prior to analysis, with one outlier being identified as 3SD, which was different from the mean and therefore excluded from analysis. Removing 2SD outliers did not alter results and these data were therefore included in the analysis. All models were estimated using restricted maximum likelihood; *p*-values were obtained using Type II Wald F tests with Kenward–Roger degrees of freedom.

OTUs (operational taxonomic units) were transformed into relative abundances as a proportion of total bacteria in the sample. Rare OTUs were removed if there was a count of less than 5 in over half of the samples at each training camp. Shannon index and Chao1 species richness were computed using the R vegan package [27], and significance testing was performed using a paired *t*-test. Non-metric multidimensional scaling (NMDS) was performed using the R vegan package and compared using analysis of variance using distance matrices (ADONIS). NMDS stress was less than 0.1 for all comparisons. The change in the relative abundance of species and relative abundance of genera were compared between pre and post intervention using a paired *t*-test with false discovery rate (FDR) correction. The change (Δ) in OTUs at species (relative abundance of species) and genus (relative abundance of genus) level was calculated as post–pre supplementation, and the Δ proportion of OTUs was compared between HCHO and LCHF diets using an unpaired *t*-test with FDR correction.

## 3. Results

### 3.1. Dietary Nitrate Content

There were no differences in the nitrate content of the LCHF and HCHO diets (*p* = 0.92). Although participants consumed more dietary nitrate during the intervention phase (213 mg/day) compared to the baseline phase (192 mg/day; *p* = 0.01), this increase was similar across both dietary groups. No differences in dietary nitrate content of the standardised breakfasts on test days were evident (*p* = 0.46). Mean nitrate contents were 299 mg/d and 307 mg/d for the HCHO diet during the baseline and intervention periods, respectively, and 291 mg/d (baseline) and 314 mg/d (intervention) for the LCHF diet.

### 3.2. Plasma Nitrate in Response to Nitrate Supplementation

**Figure 2:**
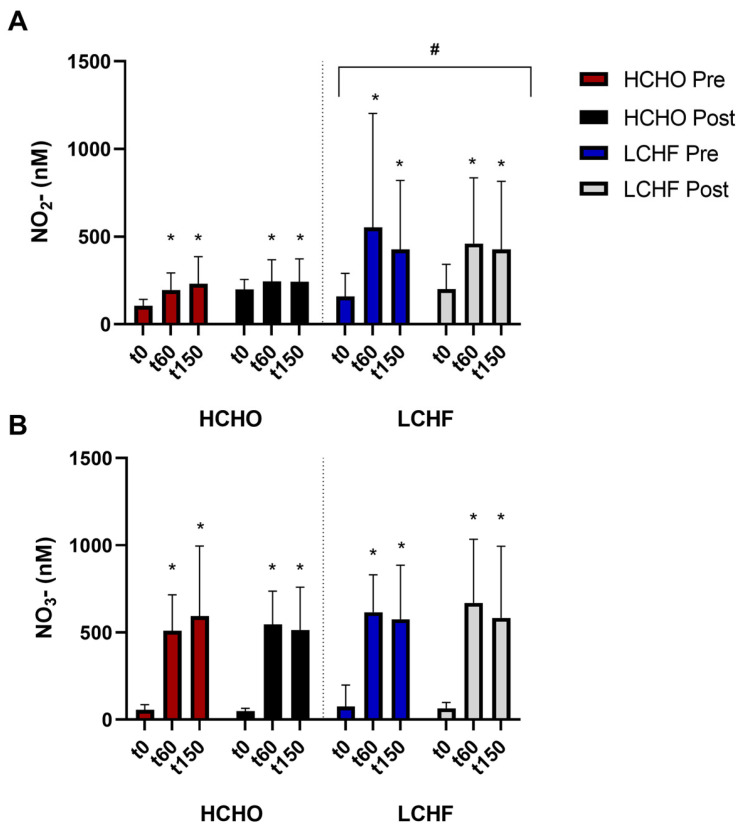
Plasma nitrite (NO_2_−) concentrations (**A**) and plasma nitrate (NO_3_−) concentrations (**B**) during baseline and intervention for athletes adhering to HCHO and LCHF. Data are presented as raw concentrations mean +/− SD. * represents a significant increase from t0. # represents a significant increase compared to HCHO.

Nitrate supplementation was associated with an increase in plasma nitrate concentration during each trial (*p* < 0.001, Figure 2A). Nitrate concentration was higher at T60 (*p* < 0.001) and T150 (*p* < 0.001) compared to T0; however, there was no difference between the latter two timepoints (*p* = 0.15). No effect of either trial (*p* = 0.85) or diet (*p* = 0.48) was evident.

### 3.3. Plasma Nitrite in Response to Nitrate Supplementation

Nitrate supplementation was associated with a significant increase in plasma nitrite concentration (*p* < 0.001, Figure 2B). Here, plasma nitrite was significantly elevated at T60 (*p* < 0.001) and T150 (*p* < 0.001) compared to T0, with no difference between T60 and T150 (*p* = 0.55). A main group effect was evident (*p* = 0.03), with plasma nitrite being higher in LCHF compared to HCHO. However, no interaction with the pre–post trial was present (*p* = 0.38), suggesting that the difference was not due to the dietary intervention, but rather the LCHF group had higher nitrate to nitrite conversion both at baseline and the following intervention.

### 3.4. Oral Microbiome Diversity

**Figure 3:**
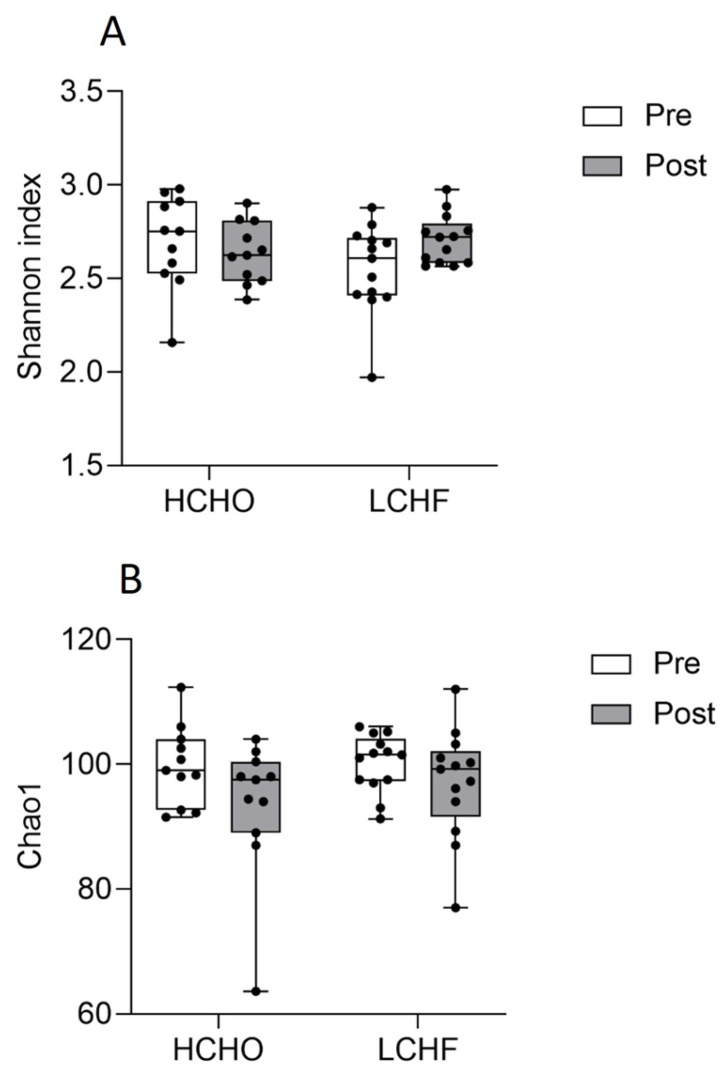
Oral bacterial diversity as represented by Shannon diversity index (**A**) and Chao1 species richness (**B**) for pre (white) and post (grey) supplementation in the HCHO and LCHF diets. Each point represents an individual oral microbiome sample. A higher score indicates higher diversity (**A**) or species richness (**B**).

**Figure 4:**
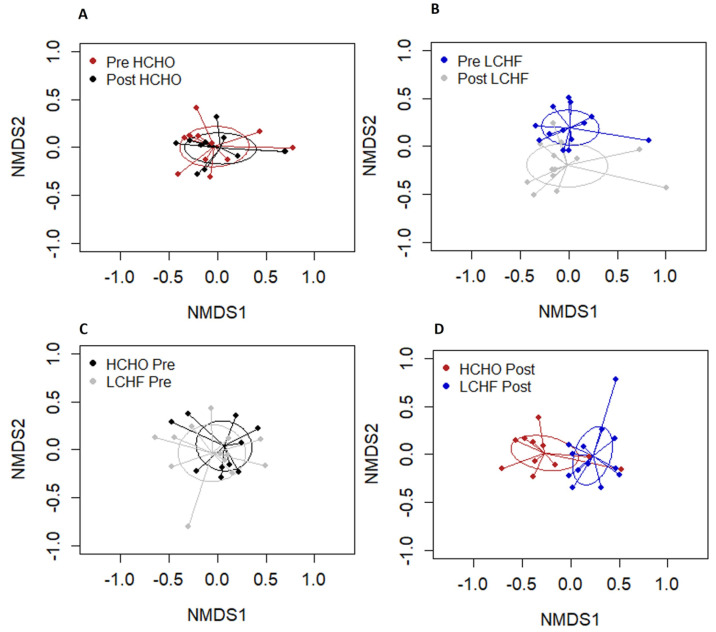
Overall salivary microbiome composition illustrated by non-metric multidimensional scaling (NMDS) analysis; HCHO pre and post supplementation (**A**), LCHF pre and post supplementation (**B**), HCHO and LCHF pre supplementation (**C**), and HCHO and LCHF post supplementation (**D**). Salivary microbiome composition was different for LCHF post intervention compared to LCHF pre intervention (**B**) and to HCHO post intervention (**D**).

There were no significant differences between pre and post HCHO and pre and post LCHF for Shannon indices (*p* = 0.192, Figure 3A) or for Chao1 species richness (*p* = 0.20, Figure 3B). NMDS revealed significant differences in sample clustering between pre and post supplementation in the LCHF diet (*p* = 0.001, Figure 4B). There was also a significant difference in sample clustering between HCHO and LCHF post supplementation (*p* = 0.001, Figure 4D). There were no significant differences in sample clustering in the pre and post supplementation HCHO diet (*p* = 0.83, Figure 4A) or between pre supplementation HCHO and LCHF (*p* = 0.97, Figure 4C).

### 3.5. Oral Microbiome Relative Abundance

**Figure 5:**
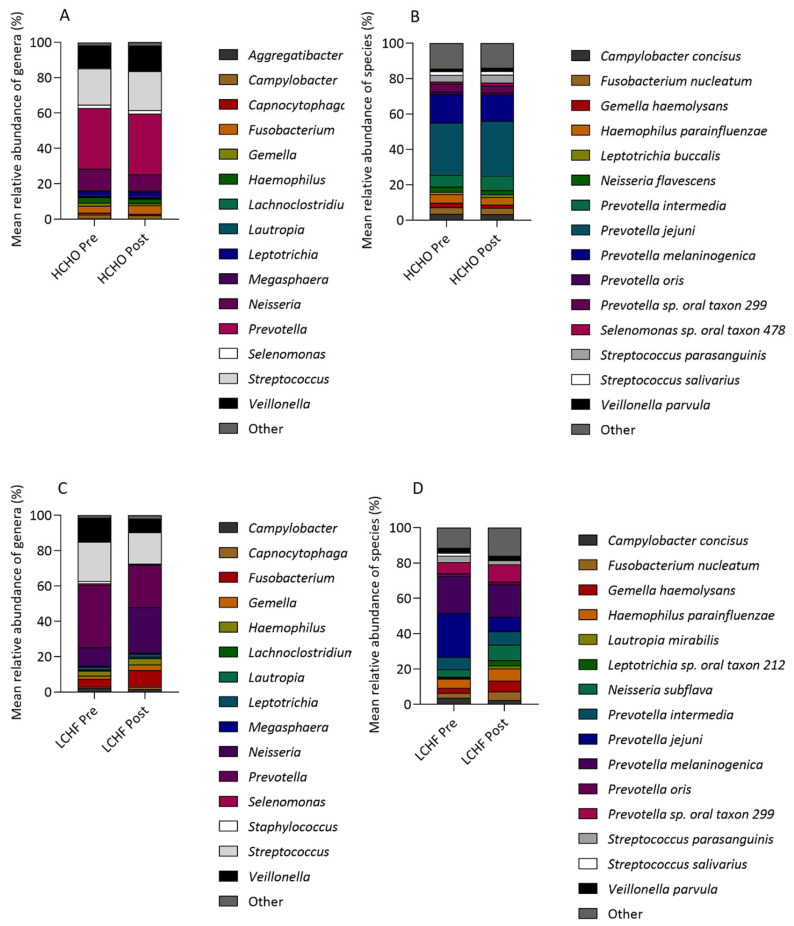
Stacked bar charts showing the mean relative abundance (%) from pre to post intervention of (**A**) HCHO genera, (**B**) HCHO species, (**C**) LCHF genera, and (**D**) LCHF species.

There were no significant changes in the oral microbiome from pre to post intervention in the HCHO diet after FDR correction. The relative abundances of four genera showed significant increases from pre to post intervention in the LCHF diet, including *Neisseria* from 10% to 25% (FDR q = 0.0002), *Fusobacterium* (5% to 9%, FDR q = 0.02), *Capnocytophaga* (0.6% to 2%, FDR q = 0.02), and *Lautropia* (0.3% to 0.7%, FDR q = 0.03). The relative abundance of two genera reduced from pre to post intervention, including *Prevotella* (34% to 23%, FDR q = 0.02) and *Veillonella* (14% to 8%, FDR q = 0.05). The species relative abundance of three OTUs increased from pre to post intervention in the LCHF diet, including *Capnocytophaga gingivalis* (0.4% to 2%, FDR q = 0.02), *Lautropia mirabilis* (1% to 2%, FDR q = 0.02), and *Leptotrichia* sp. oral taxon 212 (1% to 3%, FDR q = 0.02), whilst the relative abundance of *Prevotella jejuni* (22% to 8%, FDR q = 0.005) significantly decreased (Figure 5).

A comparison between HCHO and LCHF diets showed that there were significantly different Δ relative abundances of genera including *Neisseria* (HCHO mean Δ relative abundance, −3%; LCHF mean Δ relative abundance, 16%, FDR q < 0.0001) and species such as *p. jejuni* (HCHO mean Δ relative abundance, 2%; LCHF mean Δ relative abundance, −15, FDR q = 0.03) and *C. gingivalis* (HCHO mean Δ relative abundance, 0.03%; LCHF mean Δ relative abundance, 2%, FDR q = 0.05, Figure 5).

## 4. Discussion

This is the first study to investigate the influence of short-term (5-day) exposure to a ketogenic LCHF diet on the oral microbiome and the consequences for the response to acute dietary nitrate supplementation. Seventeen highly competitive male endurance athletes followed a supervised diet and training program for two 5-day periods involving a lead-in baseline HCHO treatment followed by an intervention (HCHO or LCHF) phase.

In this study, a LCHF diet was associated with changes to the diversity of the oral microbiome, including an increase in the relative abundance of *Neisseria* and a decrease in the relative abundance of *Prevotella* and *Veillonella* bacteria. Despite alterations in the abundance of bacteria known for their nitrate-reducing activity, both diets were associated with the expected increases in plasma nitrate and nitrite concentrations in response to nitrate (beetroot juice) supplementation [28,29]. Although this study confirmed that the LCHF diet is associated with a change in the oral microbiome [20], we conclude that sufficient nitrate-reducing activity was conserved to support the biochemical response to nitrate supplementation, a popular performance nutrition strategy [30].

### 4.1. The LCHF Diet in Sports Nutrition

The LCHF diet has been proposed as a favourable strategy for endurance athletes since it results in a substantial increase in fat oxidation during sub-maximal exercise and a reduction in reliance on the muscle’s limited carbohydrate stores [1]. Although this finding is reliably produced, even in highly trained athletes who already possess a high capacity for the use of fat as an exercise substrate, this has failed to translate to an improvement in exercise capacity [5,31] or performance [2,3]. Indeed, in the latter studies, the LCHF diet was associated with an impairment of real-life athletic performance in races involving sustained high-intensity exercise [2,3]. Performance impairment was attributed to a loss of exercise economy (i.e., an increase in the oxygen cost of exercise), which is associated with the higher oxygen cost of ATP resynthesis from fat oxidation in comparison to CHO oxidation [7], which is an outcome reported across multiple studies [2,3] and laboratories [5]. Superior exercise economy contributes to success in endurance sports and is frequently seen in elite endurance athletes due to their personal physiological characteristics and the beneficial effects of strategies such as altitude training, plyometrics, and resistance training [8,32]. 

More recently, nitrate supplementation, particularly in the form of nitrate-rich beetroot juice, has been shown to enhance exercise capacity and sports performance via an increase in exercise economy secondary to an increased NO bioavailability [33]. Although nitrate supplementation presents a possible strategy to overcome the loss of economy associated with increased reliance on fat oxidation in keto-adapted athletes, this is dependent on the presence of nitrate-reducing bacteria in the oral cavity [11]. The present study was designed to investigate the effect of exposure to a LCHF diet on the oral microbiome and its secondary effects on the response to nitrate supplementation. We confirmed that the LCHF diet is associated with changes to the oral microbiome; however, this did not impair the plasma nitrate or nitrite responses to acute nitrate supplementation.

### 4.2. The Oral Microbiome and Nitrate Metabolism

The human oral microbiome consists of an extensive and complex community of bacteria that respond to manipulations in diet and dental hygiene. The expanded Human Oral Microbiome Database (eHOMD, www.homd.org, accessed on 16 February 2023) has mapped 774 predominant oral bacterial species, which is further categorised into six major phyla. Further description of the human oral microbiome can be found in Dewhirst et al. [34].

Although the full range of activities of the oral microbiome is not currently known, specific associations with human health, physiology, metabolism, and immune response have been observed [35,36,37]. Specific oral bacteria have been associated with oral health and disease [34,38,39,40], and changes to the oral microbiome act as biomarkers for systemic disease [41] including cardiovascular disease [6,32], stroke [31], pancreatic cancer [42], preterm birth [43], Type 2 diabetes [44], and pneumonia [45].

An understanding of the function and metabolism of the oral microbiome is important for promoting optimal health. It is also relevant to athletic performance due to its relationship with the production of NO, a signalling molecule that is involved in a range of physiological processes including vasodilation, mitochondrial respiration, and skeletal muscle contraction [46,47,48]. It can be produced via two different pathways: endogenously through the L-arginine pathway in a reaction catalysed by nitric oxide synthase (NOS) or via the nitrate-nitrite-NO reduction pathway that commences with the ingestion of dietary inorganic nitrate. This latter pathway may be particularly important during high-intensity exercise because it is effective under conditions of local hypoxia and acidosis, which reduce the effectiveness of NOS.

The nitrate reduction pathway commences in the oral cavity and is dependent on the conversion of nitrate to nitrite, which is facilitated by anaerobic oral bacteria. Previous research has identified numerous bacterial genera involved in nitrate reduction in the oral cavity [12,14,15,16]. Doel et al. [14] first identified *Veillonella*, *Actinomyces*, *Rothia,* and *Staphylococcus* as the most abundant nitrate-reducing genera present in the oral cavity, with the greatest activity found with *Actinomyces* followed by *Rothia* and *Veillonella*. Hyde et al. [15] reported *Veillonella* to be the most abundant nitrate-reducing genus and identified several additional genera (*Prevotella*, *Neisseria*, and *Haemophilus*) present in higher abundance than *Actinomyces*.

The disturbance of the diversity and abundance of oral nitrate-reducing bacterial activity, such as following the use of anti-bacterial mouthwash, is associated with a blunting of the nitrate reduction pathway [13]; hence, nitrate supplementation protocols include precautions against the use of these agents. Differences in the individual oral microbiome may provide one explanation for the variability reported in response to nitrate supplementation [12].

### 4.3. Dietary-Induced Changes to the Oral Microbiome

There is evidence that the oral microbiome is malleable to dietary manipulation. Major evolutionary dietary shifts such as increased intake of processed carbohydrates [49] have been associated with significant changes to the diversity and composition of the oral microbiome. A higher intake of dietary carbohydrate favours key carbohydrate metabolising bacterial species, including *Streptococcus mutans* [50]. Other bacterial compositional changes are seen when comparing a vegan to an omnivorous diet, including an increase in *Neisseria*, *Haemophillus*, *Rothia,* and *Capnocytophaga* spp. [18], although such compositional changes are not observed in all studies [19,51]. Such changes may be related to a presumed higher intake of (nitrate-rich) vegetables in vegan/vegetarian diets, although these could also be attributed to other dietary factors known to interact with the oral microbiome such as dietary fibre and medium chain fatty acids [18]. 

There is limited literature surrounding the effect of change in dietary nitrate intake on the oral microbiome. Vanhatalo et al. [52] reported changes to the relative abundance of key nitrate-reducing bacteria following 10 days of nitrate supplementation (12 mmol/day) in young and old populations. Relative abundance increased for *Rothia* (+127%) and *Neisseria* (+351%) and decreased for *Prevotella* (−60%) and *Veillonella* (−65%). Interestingly, a functional change was reported, with increased plasma nitrite levels being associated with reduced systemic blood pressure following nitrate supplementation in older, but not younger, participants. This could be attributed to differences in individual oral microbiota and their nitrite production capacity. These findings support our result of decreased relative abundance of *Prevotella* and *Veillonella* and increased relative abundance of *Neisseria* following a 5-day LCHF diet, although Vanhatalo et al. [53] did not implement any dietary standardisation or macronutrient changes. 

There is limited prior research addressing the effect of macronutrient intake on the oral microbiome; however, our results support previous findings by Murtaza et al. [20]. These authors also reported changes to the oral microbiome following supervised intake of a ketogenic diet over 3 weeks in elite endurance athletes. Similar to our findings, Murtaza et al. [8] found a decrease in the relative abundance of *Prevotella*; however, we found an increase in *Neisseria* rather than the decrease reported by Murtaza et al. These authors also observed changes to *Haemophilus* and *Streptococcus* that we did not observe. The difference in findings speaks to the complexity of the oral microbiome and could be attributed to differences in dietary standardisation (Murtaza et al. did not account for the nitrate content of the diet) or to differences in individual oral microbiota. 

The current study adds to this work by using a previously established methodology [24] to calculate the average nitrate content of the LCHF intervention. Dietary nitrate intake was slightly higher than median nitrate intake for healthy individuals (108 mg/d) [53], which may reflect the higher overall energy intake of athletes compared to the general population. Additionally, it is noted that the LCHF diet implemented in this study was based on the “healthful” version outlined in early publications, focusing on whole foods including generous serves of green leafy vegetables to improve satiety with the otherwise energy-dense food choices [54,55]. This closely matched the vegetable content of the HCHO diet, ensuring that the diets were well matched for nitrate content and thus unlikely to influence oral microbiome changes. 

There is currently no consensus on a reference method for assessing dietary nitrate intake, which, combined with wide natural variation in the nitrate content of food, makes estimating dietary nitrate intake difficult. Factors such as the use of fertiliser, variations in soil and water nitrate content, food processing, and cooking methods [26] will all affect the nitrate content of foods, with standard deviations of mean nitrate content in published databases varying significantly [24]. 

### 4.4. LCHF Diet and Effects on Nitrate Metabolism

Following the implementation of a 5-day LCHF diet with nitrate supplementation, plasma nitrate increased similarly to previous studies of nitrate supplementation in elite athletes [29]. Higher nitrate to nitrite conversion was seen in the LCHF condition pre and post intervention compared to the HCHO group (where there were no changes to plasma nitrate post intervention), indicating that the LCHF diet did not impair nitrate conversion in these athletes despite changes to the composition and diversity of the oral microbiome.

Another study [10] that combined a 4-day non-ketogenic LCHF dietary intervention (72% fat vs. 10% fat) with nitrate supplementation (8 mmol/day potassium nitrate) reported lower plasma nitrite conversion with the high fat diet (47% increase in nitrite relative to placebo) compared to the high carbohydrate diet (88% increase relative to placebo). This was despite higher levels of plasma nitrate in the high fat diet (high fat increased by 1023% and high carbohydrate increased by 812% following nitrate supplementation compared to placebo). The authors concluded that this indicated an impairment in the nitrate-to-nitrite conversion in the high fat intervention, which may have been linked to potential changes in the oral microbiome that were not explored in the study. 

We provide strong evidence that the oral microbiome is malleable to dietary changes and that our dietary intervention altered rather than abolished nitrate-reducing species. Whilst changes to the oral microbiome could be expected to alter plasma nitrite levels, our results suggest there is enough redundancy in the microbiome to allow for oral microbial alterations and still maintain nitrite production capability. This may enable physiological changes (e.g., to systemic blood pressure or exercise performance) as a result of nitrate supplementation to still be seen.

The magnitude of alteration to the microbiome may determine the functional change following nitrate supplementation. For example, the use of antiseptic mouthwash markedly attenuates the rise in plasma nitrite following nitrate supplementation [56], with this response being evident when using both strong and weak mouthwash [57]. The use of antiseptic mouthwash causes large shifts in the oral microbiome [51,58], whereas we saw more subtle changes to the bacterial community following dietary intervention. 

This raises the possibility that although the relative abundance of key nitrate-reducing bacteria was significantly reduced in our study, there was still sufficient bacterial diversity remaining to enable other nitrite-producing bacterial species to increase their own nitrite production activity, thus preventing changes to plasma nitrite levels. Another possibility is that the total number of available nitrite-producing bacteria may have remained high enough to continue nitrite production at baseline levels, with previous studies also identifying that total abundance, as well as diversity, may be key to salivary nitrite production [16]. Of note is that our microbiome analysis did not quantify total bacterial mass, only relative abundance. Preservation of nitrate conversion despite changes to the diversity and abundance of the key nitrite-producing bacteria may be related to complexities within the oral microbiome network [59] and warrants further research.

### 4.5. Limitations

The small subject sample size is acknowledged as a potential limitation of the study, and this study was not powered to enable further network analyses on the oral microbiome interactions with physiological variables. However, a larger sample size was not possible given the elite training status of our athletes, and further research is warranted to compare ‘microbiome-physical performance’ interaction in athletic and non-athletic populations.

We used 16S rRNA sequencing to assess changes in the relative abundances of oral bacteria subsequent to dietary interventions. While this method does not enable the identification of functional genes within the bacterial genome, it offers a cost-effective tool with appropriate sensitivity to capture whole-community shifts in diversity and relative abundances [60] such as those seen in our study, as well as enabling comparison with previous research reporting changes in the oral microbiome assessed by 16S sequencing following dietary interventions [16,52].

## 5. Conclusions

We aimed to describe changes in the oral microbiome consequent to a combined tightly controlled standardised LCHF dietary and nitrate supplementation intervention. Our study is one of very few investigating diet and oral microbiome interaction in elite athletes and the first to include nitrate supplementation outcomes. We found that nitrate supplementation increased plasma nitrate and nitrite levels independent of dietary condition and despite a significant change to oral bacterial diversity and relative abundance of nitrate-reducing bacteria observed in the LCHF condition. Our study provides early evidence that even when the oral microbiome is altered by a LCHF diet, some capacity to maintain nitrite production remains.

## Data Availability

The data presented in this study are available on request from the corresponding author. The data are not publicly available due to privacy restrictions.
